# From net effects to configurational pathways: a dual examination of employee innovative behavior through integrated SEM-fsQCA framework

**DOI:** 10.3389/fpsyg.2026.1729200

**Published:** 2026-02-18

**Authors:** Yongji Jiao, Hai Cheng, Xinyi Huang, Jieyu Ouyang, Qishu Li, Ruirui Wang

**Affiliations:** School of Management, Nanjing University of Posts and Telecommunications, Nanjing, China

**Keywords:** employee trait mindfulness, innovative behavior, work thriving, organizational resilience, digital leadership, fuzzy-set qualitative comparative analysis

## Abstract

**Introduction:**

While conventional structural equation modeling (SEM) emphasizes net effects between variables, it inadequately captures the multiple equifinal pathways underlying employee innovative behavior. Drawing on componential creativity theory and social cognitive theory, this study advances a methodological triangulation approach by integrating SEM with fuzzy-set qualitative comparative analysis (fsQCA) to examine the mechanisms through which trait mindfulness influences innovative behavior.

**Methods:**

We analyzed data from 297 employees in digitally transforming organizations, employing both SEM and fsQCA to test symmetric causal relationships and identify asymmetric configurational patterns.

**Results:**

SEM findings reveal that trait mindfulness affects innovative behavior through work flourishing (full mediation) and organizational resilience (partial mediation), with digital leadership exerting a negative moderating effect. fsQCA identifies three equifinal configurations leading to high innovative behavior, with the “high mindfulness × high resilience” configuration emerging as the dominant pathway.

**Discussion:**

This study demonstrates the methodological complementarity of SEM-fsQCA integration: whereas SEM elucidates symmetric causal relationships and average effects, fsQCA unveils asymmetric configurational patterns and necessary/sufficient conditions, providing a more comprehensive understanding of the complex mechanisms driving employee innovation.

## Introduction

Employee innovative behavior has emerged as a critical determinant of organizational competitiveness in contemporary business environments (Yuan and Woodman, 2010; Wang et al., 2022). While trait mindfulness has been identified as a promising psychological resource for promoting innovation (Reb et al., 2014; Hülsheger et al., 2013), the mechanisms through which it influences innovative behavior remain inadequately understood. This knowledge gap is particularly salient given the digital transformation context, where organizations must simultaneously balance present-focused mindfulness practices with future-oriented digital leadership approaches.

Current research on mindfulness and innovation exhibits three critical limitations. First, existing studies have produced inconsistent findings, with effect sizes varying substantially across contexts, suggesting that simple linear relationships may inadequately capture the complexity of innovation processes. Second, theoretical frameworks have largely overlooked the potential for multiple equifinal pathways to innovation, wherein different combinations of individual and organizational factors produce equivalent outcomes through distinct mechanisms. Third, methodological approaches have predominantly relied on variable-centered analyses that assume causal uniformity, potentially masking configurational patterns that operate simultaneously in organizational settings.

This study addresses these limitations by examining how trait mindfulness influences employee innovative behavior through dual mediating pathways—thriving at work and organizational resilience—with digital leadership as a boundary condition. We employ a dual-method approach combining structural equation modeling (SEM) to test hypothesized relationships and fuzzy-set qualitative comparative analysis (fsQCA) to identify multiple sufficient configurations for high innovation. This methodological integration enables examination of both average causal effects and context-dependent pathways.

The research contributes to organizational psychology literature in three ways. Theoretically, we demonstrate that mindfulness and digital leadership operate through configurational rather than simple linear effects, extending social cognitive theory’s understanding of triadic reciprocal determinism (Bandura, 1986). Methodologically, we provide empirical evidence that innovation formation follows multiple generative mechanisms, advancing componential creativity theory (Amabile, 1983) beyond assumptions of causal uniformity. Practically, we offer diagnostic configuration maps that enable organizations to identify optimal innovation pathways based on their specific resource portfolios, shifting innovation management from prescriptive universalism toward contextualized strategy formulation.

## Theoretical foundation and research hypotheses

**Figure 1:**
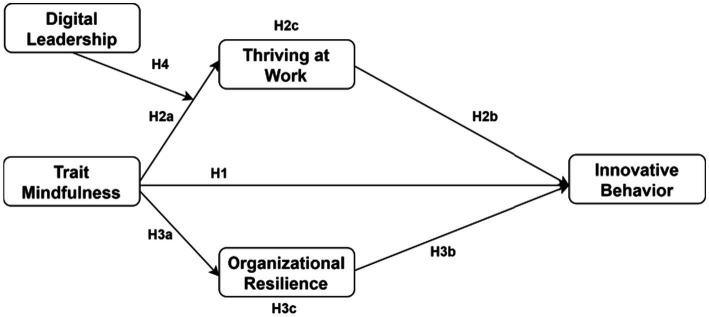
Presents the integrated research model examining these multilevel relationships.

This study integrates Amabile’s (1983) componential theory of creativity with Bandura’s (1986) social cognitive theory to explain how trait mindfulness influences employee innovative behavior. Componential creativity theory posits that innovation emerges from dynamic interactions among domain-relevant skills, creativity-relevant processes, and task motivation. This framework aligns conceptually with trait mindfulness, which enhances attention regulation, cognitive awareness, and open-mindedness—key components of creativity-relevant processes. Social cognitive theory’s triadic reciprocal determinism further suggests that behavior results from continuous reciprocal interactions among personal factors, behavioral patterns, and environmental influences. Applied to the innovation context, this implies that individual psychological resources such as mindfulness do not operate in isolation but rather interact with organizational conditions and leadership environments to shape innovative outcomes (Figure 1).

Traditional variable-centered approaches assume homogeneous causal effects, wherein trait mindfulness uniformly influences innovation through consistent pathways across all contexts. However, componential creativity theory suggests that innovation emerges from complex interactions among multiple components, implying the possibility of equifinality—that different configurations of individual and organizational factors may produce equivalent innovative outcomes through distinct mechanisms. This theoretical consideration necessitates methodological approaches capable of capturing configurational complexity. Therefore, while we develop directional hypotheses based on theoretical reasoning to be tested through structural equation modeling, we also recognize that multiple sufficient pathways to high innovation may exist, which we explore through fuzzy-set qualitative comparative analysis.

### Trait mindfulness and innovative behavior

Trait mindfulness refers to stable dispositional tendencies toward maintaining present-moment awareness characterized by openness, curiosity, and acceptance of experiences without judgment (Brown and Ryan, 2003). Research has demonstrated consistent positive associations between trait mindfulness and creative performance across organizational contexts. Mindfulness has been found to enhance both creative thinking and creative self-efficacy through improved cognitive flexibility (Good et al., 2016). Similarly, trait mindfulness has been shown to promote innovative work behavior by strengthening occupational identity and facilitating thriving at work (Reb et al., 2014).

The theoretical mechanisms linking trait mindfulness to innovative behavior operate through three primary pathways. First, mindfulness enhances attentional focus and awareness, enabling employees to identify novel opportunities and recognize problems that others might overlook (Langer and Moldoveanu, 2000). Second, mindfulness improves emotional regulation capacity, allowing individuals to maintain psychological equilibrium during the inherently uncertain innovation process and persist despite setbacks (De Jong and Den Hartog, 2010). Third, the non-judgmental observation characteristic of mindfulness helps individuals break free from conventional thinking patterns and cognitive rigidity, facilitating the generation of novel ideas (Baer and Oldham, 2006). Drawing on this theoretical foundation and empirical evidence, we hypothesize:

H1: Employee trait mindfulness is positively related to innovative behavior.

### Mediating role of thriving at work

Thriving at work represents a psychological state characterized by the joint experience of vitality—a sense of energy and aliveness—and learning—the acquisition and application of knowledge and skills (Spreitzer et al., 2005). Research has established thriving as a critical mediating mechanism linking individual resources and contextual factors to behavioral outcomes including innovation (Kleine et al., 2019; Alikaj et al., 2021). Theoretically, thriving serves as a proximal motivational state that translates distal resources such as trait mindfulness into concrete innovative actions.

Trait mindfulness is expected to enhance thriving through dual pathways. First, mindfulness reduces self-criticism and rumination while enhancing positive emotional experiences, thereby increasing vitality (Paterson et al., 2014). Employees who maintain non-judgmental awareness experience greater psychological energy and enthusiasm for their work. Second, mindfulness fosters cognitive openness and curiosity, facilitating knowledge absorption and learning from experiences (Bostock et al., 2019). By maintaining present-moment awareness, mindful employees more effectively notice learning opportunities and integrate new information. In turn, thriving employees exhibit heightened intrinsic motivation and psychological resources that enable them to explore innovative solutions and take initiative in implementing new ideas (Carmeli and Spreitzer, 2009). The combination of vitality and learning creates an optimal psychological state for engaging in the effortful and uncertain process of innovation. Based on this theoretical reasoning, we propose:

H2a: Trait mindfulness positively influences thriving at work.

H2b: Thriving at work positively influences innovative behavior.

H2c: Thriving at work mediates trait mindfulness-innovative behavior relationship.

### Mediating role of organizational resilience

Organizational resilience refers to an organization’s capacity to anticipate, prepare for, respond to, and adapt to incremental change and sudden disruptions in order to survive and prosper (Lengnick-Hall et al., 2011). Research has demonstrated that organizational resilience serves as a critical mediating mechanism linking individual-level resources to organizational outcomes (Duchek, 2020; Celestin and Vanitha, 2020). In the context of innovation, organizational resilience provides the supportive infrastructure and psychological safety necessary for employees to take risks and experiment with novel approaches.

Trait mindfulness at the individual level may aggregate to influence organizational resilience through two mechanisms. First, when employees exhibit higher trait mindfulness, they demonstrate greater adaptability and cognitive flexibility when facing challenges, which collectively enhances the organization’s capacity to respond to environmental changes (Liu et al., 2025). Second, mindfulness reduces excessive fear of failure and builds confidence in handling uncertainty, creating a cultural foundation that supports organizational learning from setbacks rather than avoidance (Majeed et al., 2023). Organizations characterized by strong resilience, in turn, provide supportive environments marked by psychological safety, resource availability, and tolerance for failure—conditions essential for employee innovation (Sutcliffe et al., 2016; Kaiyun et al., 2025). When employees perceive their organization as resilient, they feel more confident in proposing and implementing innovative ideas despite inherent risks. Therefore, we hypothesize:

H3a: Trait mindfulness positively influences organizational resilience.

H3b: Organizational resilience positively influences innovative behavior.

H3c: Organizational resilience mediates trait mindfulness-innovative behavior relationship.

### Moderating role of digital leadership

Digital leadership encompasses leadership behaviors and competencies specifically adapted for digital environments, including the ability to leverage digital technologies, facilitate digital transformation, and guide employees through technology-driven organizational changes (Wang et al., 2022). Research has demonstrated that digital leadership significantly influences employee attitudes and behaviors in digitally transforming organizations (Mihardjo et al., 2019; Yongyue and Lihua, 2024). However, the moderating role of digital leadership in the mindfulness-innovation relationship remains theoretically ambiguous, as digital leadership may produce both enabling and constraining effects.

On one hand, digital leadership may strengthen the positive effects of trait mindfulness on innovative behavior by providing technological infrastructure, digital tools, and data-driven resources that amplify employees’ capacity to transform mindful awareness into concrete innovative actions (Fredrickson, 2001). Digital leaders who effectively communicate vision and provide technological support may create conditions under which mindful employees can more efficiently identify opportunities and implement innovations. On the other hand, excessive digital leadership intervention may undermine employee autonomy and increase performance pressure, potentially offsetting the psychological benefits of mindfulness (Tigre et al., 2023). When digital leadership becomes too controlling or demands continuous connectivity, it may diminish the psychological space necessary for mindfulness to facilitate creative thinking. Given these competing theoretical perspectives, we propose an exploratory hypothesis.

H4: Digital leadership moderates the trait mindfulness-innovative behavior relationship

## Research design

### Research sample and data collection

We recruited participants from enterprises undergoing digital transformation across four industry sectors: internet technology, advanced manufacturing, financial services, and telecommunications. To ensure sample appropriateness, we established three inclusion criteria: participants must have at least one year of organizational tenure to possess sufficient familiarity with organizational practices and culture, be currently employed in organizations actively engaged in digital transformation initiatives, and provide voluntary informed consent for participation. These criteria ensured that respondents had adequate experience to meaningfully evaluate both individual psychological states and organizational characteristics relevant to digital transformation contexts.

Data collection occurred between March and June 2025 through a multi-channel approach combining online and offline survey administration. We distributed questionnaires through three primary channels: professional survey platforms, corporate human resources networks with organizational approval, and targeted social media professional groups. Prior to formal data collection, we conducted expert review with three organizational psychology professors and pilot testing with 30 employees to optimize questionnaire clarity and content validity. The pilot test results indicated satisfactory reliability across all measures (Cronbach’s *α* > 0.70) and led to minor wording adjustments to improve item comprehension.

To address common method bias concerns, we implemented several procedural safeguards. First, we incorporated reverse-coded items within each measurement scale to reduce acquiescence response patterns. Second, we embedded attention check items throughout the questionnaire to identify careless responding. Third, we assured participants of response confidentiality and anonymity, and emphasized that participation was voluntary with no consequences for non-participation or withdrawal. Fourth, we provided clear instructions that there were no right or wrong answers and encouraged honest responses. Following data collection, we rigorously screened responses by removing questionnaires with missing data exceeding 10%, failed attention checks, or completion times suggesting insufficient engagement (less than 5 min or exceeding 40 min).

The final sample comprised 297 valid responses from an initial distribution of 432 questionnaires, yielding an effective response rate of 68.75%. Sample characteristics are presented in Table 1.

**Table 1:** Descriptive statistics of formal survey data.

Variable	Category	Frequency	Percentage
Gender	Male	122	41.10%
	Female	175	58.90%
Age	18–25	46	15.50%
	26–30	92	31.00%
	31–35	72	24.20%
	36–40	43	14.50%
	40–50	33	11.10%
	51–60	11	3.70%
Education	High school or below	9	3.00%
	College diploma	30	10.10%
	Bachelor’s degree	166	55.90%
	Master’s degree	79	26.60%
	Doctoral degree or above	13	4.40%
Enterprise type	State-owned	87	29.30%
	Private	150	50.50%
	Foreign-invested	32	10.80%
	Mixed ownership	17	5.70%
	Other	11	3.70%
Enterprise size	<100 employees	71	23.90%
	100–500	130	43.80%
	500–1,000	51	17.20%
	≥1,000	45	15.20%
Industry	Manufacturing	54	18.20%
	Internet	73	24.60%
	Finance	40	13.50%
	Construction/real estate	24	8.10%
	Wholesale/retail	30	10.10%
	Food service	10	3.40%
	Education/training	28	9.40%
	Business services	13	4.40%
	Other	25	8.40%
Position level	Frontline staff	135	45.50%
	Frontline management	108	36.40%
	Middle management	42	14.10%
	Senior management	12	4.00%
Digital transformation duration	Not started	0	0%
	1–5 years	207	69.70%
	6–10 years	66	22.20%
	>10 years	24	8.10%

### Variable measurement

The questionnaire consisted of three main sections: an introductory section explaining research purposes and privacy protection to enhance response authenticity, a measurement section assessing the five core constructs, and a demographic information section providing stratification variables for subsequent analysis. All substantive measures employed established scales with demonstrated psychometric properties in organizational research contexts. Responses were recorded using 5-point Likert scales ranging from 1 (strongly disagree) to 5 (strongly agree), with higher scores indicating greater levels of the focal construct.

Trait mindfulness was measured using the 15-item Mindful Attention Awareness Scale developed by Brown and Ryan (2003). This scale assesses individual dispositional tendencies toward present-moment awareness and attention to ongoing experiences. Representative items include “I find it difficult to stay focused on what’s happening in the present” (reverse-coded) and “I tend to walk quickly to get where I’m going without paying attention to what I experience along the way” (reverse-coded). The scale has been widely validated across diverse cultural and organizational contexts. In the current sample, the measure demonstrated excellent internal consistency reliability (Cronbach’s *α* = 0.942) and sampling adequacy (Kaiser-Meyer-Olkin measure = 0.961).

Digital leadership was assessed using a 6-item scale adapted from Zeike et al. (2019), which captures leadership behaviors and competencies specific to digital transformation contexts. The scale measures leaders’ abilities to facilitate digital initiatives, communicate digital vision, and support employees through technology-driven changes. Sample items include “My leader effectively communicates the vision for digital transformation” and “My leader provides adequate support for using new digital technologies.” This measure demonstrated acceptable internal consistency (Cronbach’s *α* = 0.819) and sampling adequacy (KMO = 0.864) in the present study.

Thriving at work was measured using the 10-item scale developed by Porath et al. (2012), which assesses the two core dimensions of thriving: vitality and learning. The vitality subscale captures feelings of energy and aliveness at work (e.g., “I feel alive and vital”), while the learning subscale measures the sense of continuous improvement and skill development (e.g., “I am learning and developing as a person”). The complete scale demonstrated good internal consistency (Cronbach’s *α* = 0.882) and adequate sampling adequacy (KMO = 0.917).

Organizational resilience was assessed using a 15-item scale adapted from Burnard and Bhamra (2011), encompassing three dimensions: adaptive capacity, anticipation capability, and situational awareness. The adaptive capacity dimension measures organizational flexibility in responding to changes (e.g., “Our organization quickly adjusts strategies when facing unexpected challenges”). The anticipation capability dimension assesses proactive planning and risk management (e.g., “Our organization systematically identifies potential risks”). The situational awareness dimension captures organizational consciousness of environmental changes (e.g., “Our organization maintains awareness of changes in the external environment”). The overall scale exhibited excellent reliability (Cronbach’s *α* = 0.917) and sampling adequacy (KMO = 0.951).

Innovative behavior was measured using the 6-item scale developed by Scott and Bruce (1994), which assesses employees’ engagement in innovation-related activities including idea generation, championing, and implementation. Representative items include “I generate creative ideas” and “I promote and champion ideas to others.” This widely used measure has demonstrated validity across numerous organizational studies. In the current sample, it showed good internal consistency (Cronbach’s *α* = 0.830) and adequate sampling adequacy (KMO = 0.863).

All measurement scales demonstrated satisfactory psychometric properties. Internal consistency reliability coefficients exceeded the conventional threshold of 0.80, indicating that items within each scale reliably measured their respective constructs. KMO measures of sampling adequacy all exceeded 0.80, and Bartlett’s tests of sphericity were significant at *p* < 0.001, confirming that the correlation matrices were suitable for factor analysis. These results provide confidence in the reliability and construct validity of the measurements used in this study.

## Empirical analysis

### Common method Bias test

This study employed Harman’s single-factor test for common method bias. Unrotated principal component factor analysis of all study variables extracted 8 factors with eigenvalues greater than 1, cumulatively explaining 56.071% of total variance. The first factor explained 35.755% of variance, below the 40% threshold, indicating no serious common method bias.

### Confirmatory factor analysis

Confirmatory factor analysis was conducted for all five variables to assess discriminant validity. Five nested models were tested in Mplus: one-factor model (all variables combined), two-factor model (trait mindfulness + digital leadership; thriving at work + organizational resilience + innovative behavior), three-factor model (trait mindfulness; digital leadership + thriving at work; organizational resilience + innovative behavior), four-factor model (trait mindfulness; digital leadership; thriving at work + organizational resilience; innovative behavior), and five-factor model (all variables separate).

Results show that model fit improved with increasing factor numbers. The five-factor model demonstrated the best fit with all indices within acceptable ranges: RMSEA = 0.041, CFI = 0.920, TLI = 0.916, SRMR = 0.057, confirming good discriminant validity among the five variables. Results are presented in Table 2.

**Table 2:** Confirmatory factor analysis results for multi-factor models (*N* = 297).

Model	χ^2^	df	χ^2^/df	RMSEA	CFI	TLI	SRMR
Ideal fit indices			<3	<0.08	>0.9	>0.9	<0.08
Five-factor model	1,899.429	1,264	1.502***	0.041	0.92	0.916	0.057
Four-factor model	1,963.883	1,268	1.548***	0.043	0.912	0.908	0.059
Three-factor model	2,044.959	1,271	1.608***	0.045	0.903	0.898	0.059
Two-factor model	2,752.442	1,273	2.162***	0.063	0.814	0.806	0.145
One-factor model	4,530.271	1,274	3.555***	0.093	0.59	0.574	0.145

***p < 0.001.

Model Specifications:

Five-factor: Trait mindfulness, digital leadership, thriving at work, organizational resilience, innovative behavior (all separate).

Four-factor: Trait mindfulness, digital leadership, thriving + organizational resilience, innovative behavior.

Three-factor: Trait mindfulness, digital leadership + thriving, organizational resilience + innovative behavior.

Two-factor: Trait mindfulness + digital leadership, thriving + organizational resilience + innovative behavior.

One-factor: All variables combined

### Correlation analysis

Correlation analysis was conducted using SPSS Statistics 26 to examine relationships among the five variables. Results are shown in Table 3.

**Table 3:** Correlation analysis results (*N* = 297).

Variable	1	2	3	4	5
1. Trait mindfulness	1				
2. Digital leadership	0.523**	1			
3. Thriving at work	0.686**	0.694**	1		
4. Organizational resilience	0.649**	0.756**	0.827**	1	
5. Innovative behavior	0.559**	0.670**	0.779**	0.767**	1

**p < 0.01.

All five variables demonstrated significant positive correlations (*p* < 0.01). Specifically, trait mindfulness significantly correlated with digital leadership (*r* = 0.523**), thriving at work (*r* = 0.686**), organizational resilience (*r* = 0.649**), and innovative behavior (*r* = 0.559**), providing initial support for trait mindfulness’s potential positive effects on employee work states and behaviors.

Digital leadership showed strong positive correlations with thriving at work (*r* = 0.694**), organizational resilience (*r* = 0.756**), and innovative behavior (*r* = 0.670**), suggesting its importance in promoting these outcomes. Thriving at work and organizational resilience demonstrated a strong correlation (*r* = 0.827**), while both strongly correlated with innovative behavior (*r* = 0.779** and *r* = 0.767**, respectively), providing preliminary support for their potential mediating roles.

These correlation results provide foundational support for subsequent hypothesis testing, though causal relationships require further verification through structural equation modeling.

### Hypothesis testing

#### Testing 1. Regression analysis

We conducted hierarchical multiple regression analysis to test the hypothesized relationships, with results presented in Table 4. The analysis followed a systematic approach to examine direct effects, mediating pathways, and moderating influences of the focal variables.

**Table 4:** Regression analysis results (*N* = 297).

Model	Dependent variable	Independent variable	*β*	*t*-value	*R* ^2^	Adjusted-*R*^2^	*F*-value
1	Innovative behavior	Trait mindfulness	0.559	11.567***	0.312	0.31	133.796***
2	Thriving at work	Trait mindfulness	0.686	16.192***	0.471	0.469	262.177***
3	Organizational resilience	Trait mindfulness	0.649	14.640***	0.421	0.419	214.321***
4	Innovative behavior	Thriving at work	0.779	21.331***	0.607	0.605	455.015***
5	Innovative behavior	Organizational resilience	0.767	20.555***	0.589	0.587	422.503***

***p < 0.001.

Model 1 tested the direct effect of trait mindfulness on innovative behavior (Hypothesis 1). Results indicated that trait mindfulness exerted a significant positive effect on innovative behavior (*β* = 0.559, *t* = 11.567, *p* < 0.001), accounting for 31.2% of the variance in innovative behavior (*R*^2^ = 0.312). This substantial effect size demonstrates that employees with higher dispositional mindfulness exhibit significantly greater innovative behavior, providing strong support for Hypothesis 1.

To examine the mediating role of thriving at work, we conducted a series of regression analyses following established mediation testing procedures. Model 2 tested whether trait mindfulness significantly predicted thriving at work (Hypothesis 2a). The analysis revealed that trait mindfulness had a substantial positive effect on thriving at work (*β* = 0.686, *t* = 16.192, *p* < 0.001), explaining 47.1% of the variance in thriving (*R*^2^ = 0.471). This strong relationship indicates that mindful employees experience significantly higher levels of vitality and learning at work, thus supporting Hypothesis 2a. Model 4 subsequently examined whether thriving at work predicted innovative behavior (Hypothesis 2b). Results demonstrated that thriving at work significantly and positively influenced innovative behavior (*β* = 0.779, *t* = 21.331, *p* < 0.001), accounting for 60.7% of the variance (*R*^2^ = 0.607). The magnitude of this effect suggests that thriving represents a critical proximal psychological state that drives innovation, thereby supporting Hypothesis 2b. The substantial explained variance and strong coefficients across these models provide compelling evidence that thriving at work serves as an important mediating mechanism linking trait mindfulness to innovative behavior, offering preliminary support for Hypothesis 2c.

We employed a parallel analytical approach to test the mediating role of organizational resilience. Model 3 examined whether trait mindfulness predicted organizational resilience (Hypothesis 3a). The regression analysis demonstrated that trait mindfulness significantly and positively affected organizational resilience (*β* = 0.649, *t* = 14.640, *p* < 0.001), explaining 42.1% of the variance (*R*^2^ = 0.421). This finding indicates that when employees exhibit higher mindfulness, they perceive their organizations as more resilient and adaptive, thus supporting Hypothesis 3a. Model 5 tested whether organizational resilience predicted innovative behavior (Hypothesis 3b). Results revealed that organizational resilience exerted a significant positive effect on innovative behavior (*β* = 0.767, *t* = 20.555, *p* < 0.001), accounting for 58.9% of the variance (*R*^2^ = 0.589). This strong relationship suggests that perceived organizational resilience creates a supportive context that encourages employees to engage in innovative activities, thereby supporting Hypothesis 3b. Together with the established direct relationship between trait mindfulness and innovative behavior, these findings fulfill the conditions for mediation, providing preliminary support for Hypothesis 3c that organizational resilience mediates the trait mindfulness-innovative behavior relationship.

#### Testing 2. Mediation effect testing

To precisely examine the mediating role of thriving at work in the relationship between trait mindfulness and innovative behavior, this study employed Bootstrap method (PROCESS macro) for mediation analysis. Results are shown in Table 5.

**Table 5:** Mediation analysis results for thriving at work.

Effect type	Effect value	Standard error	*t*	*p*	95% CI lower	95% CI upper
Total effect	0.6098	0.0527	11.567	0	0.506	0.7135
Direct effect	0.0501	0.0548	0.9144	0.3613	−0.0577	0.158
Indirect effect	0.5597	0.0677	–	–	0.4205	0.6829

Bootstrap mediation analysis results show that the total effect of trait mindfulness on innovative behavior was significant (*β* = 0.6098, *t* = 11.567, *p* < 0.001). However, after controlling for thriving at work, the direct effect of trait mindfulness on innovative behavior became non-significant (*β* = 0.0501, *t* = 0.9144, *p* = 0.3613, 95% CI: [−0.0577, 0.1580]), with the confidence interval including 0. The indirect effect through thriving at work was significant (*β* = 0.5597, 95% CI: [0.4205, 0.6829]), with the confidence interval excluding 0. These results indicate that thriving at work plays a complete mediating role in the relationship between trait mindfulness and innovative behavior.

Hypothesis H2c is supported: Thriving at work mediates the relationship between trait mindfulness and innovative behavior.

To examine the mediating role of organizational resilience in the relationship between trait mindfulness and innovative behavior, this study also employed Bootstrap method for analysis. Results are shown in Table 6.

**Table 6:** Mediation analysis results for organizational resilience.

Effect type	Effect value	Standard error	*t*	*p*	95% CI lower	95% CI upper
Total effect	0.6098	0.0527	11.567	0	0.506	0.7135
Direct effect	0.1146	0.0532	2.1535	0.0321	0.0099	0.2194
Indirect effect	0.4952	0.0576	–	–	0.3821	0.6067

Bootstrap mediation analysis results show that the total effect of trait mindfulness on innovative behavior was significant (*β* = 0.6098, *t* = 11.567, *p* < 0.001). After controlling for organizational resilience, the direct effect of trait mindfulness on innovative behavior remained significant though weakened (*β* = 0.1146, *t* = 2.1535, *p* = 0.0321, 95% CI: [0.0099, 0.2194]), with the confidence interval excluding 0. The indirect effect through organizational resilience was significant (*β* = 0.4952, 95% CI: [0.3821, 0.6067]), with the confidence interval excluding 0. These results indicate that organizational resilience plays a partial mediating role in the relationship between trait mindfulness and innovative behavior.

Hypothesis H3c is supported: Organizational resilience mediates the relationship between trait mindfulness and innovative behavior.

#### Testing 3. Moderation effect testing

To examine the moderating role of digital leadership in the relationship between trait mindfulness and thriving at work, this study employed PROCESS macro for moderation analysis. Results are shown in Table 7.

**Table 7:** Moderation analysis results for digital leadership.

Digital leadership	Moderation effect	Bootstrap SE	95% CI lower	95% CI upper
−0.6833	0.379	0.0466	0.2882	0.4713
0	0.2923	0.055	0.1861	0.3993
0.6833	0.2057	0.0714	0.0652	0.34
Moderated mediation	−0.1268	0.0362	−0.1995	−0.0606

Analysis results show that digital leadership significantly moderates the relationship between trait mindfulness and thriving at work. Specifically, under different levels of digital leadership, the conditional effects of trait mindfulness on thriving at work varied. When digital leadership was low (−0.6833), the effect of trait mindfulness on thriving at work was 0.3790 (95% CI: [0.2882, 0.4713]); at moderate levels of digital leadership (0), the effect was 0.2923 (95% CI: [0.1861, 0.3993]); when digital leadership was high (0.6833), the effect decreased to 0.2057 (95% CI: [0.0652, 0.3400]).

These results indicate that as digital leadership levels increase, the positive effect of trait mindfulness on thriving at work gradually weakens. The moderated mediation effect was −0.1268 (95% CI: [−0.1995, −0.0606]), with the confidence interval excluding 0, indicating that digital leadership indirectly moderates the effect of trait mindfulness on innovative behavior by moderating the relationship between trait mindfulness and thriving at work, with this moderation effect being negative.

Hypothesis H4 is supported: Digital leadership has a negative moderating effect on the relationship between trait mindfulness and innovative behavior.

### fsQCA configuration analysis

#### Analysis 1. Data calibration

In fsQCA, data calibration converts raw data into fuzzy membership values between 0 and 1. This study employed the indirect method, setting three anchor points: full membership (95%), crossover point (50%), and full non-membership (5%). Anchor point thresholds were calculated using SPSS 26, then calibration conversion was completed using fsQCA software’s Calibrate function. The calibration anchor points for trait mindfulness, digital leadership, thriving at work, organizational resilience, and innovative behavior are shown in Table 8.

**Table 8:** Calibration anchor points.

Variable	Full membership (95%)	Crossover point (50%)	Full non-membership (5%)
Trait mindfulness	4.4	3.07	1.6
Digital leadership	4.67	4	2.47
Thriving at work	4.6	4.2	2.69
Organizational resilience	4.53	4	2.53
Innovative behavior	4.67	4	2.48

#### Analysis 2. Necessary condition analysis

In necessary condition analysis, consistency should reach 0.9 to be considered a strict necessary condition. Generally, when a condition’s consistency score approaches or exceeds 0.85, it can be regarded as a necessary condition for the outcome. When coverage approaches or exceeds 0.75, it indicates these conditions have strong explanatory power for the outcome.

This study’s fsQCA analysis reveals that organizational resilience, digital leadership, and thriving at work, while not reaching strict necessary condition thresholds, all demonstrate near-necessary characteristics, indicating these organizational factors play significant roles in promoting high employee innovative behavior. Among these, organizational resilience shows the most prominent coverage, suggesting the highest correlation with high innovative behavior. Results are shown in Table 9.

**Table 9:** Necessary condition analysis.

Condition variable	High innovative behavior		Non-high innovative behavior	
	Consistency	Coverage	Consistency	Coverage
Trait mindfulness	0.654774	0.704139	0.66221	0.549824
~Trait mindfulness	0.581385	0.690329	0.643663	0.590082
Digital leadership	0.815453	0.82615	0.608268	0.475791
~Digital leadership	0.482578	0.614729	0.777743	0.764915
Thriving at work	0.815334	0.854917	0.580989	0.470346
~Thriving at work	0.494869	0.604695	0.820788	0.774351
Organizational resilience	0.834368	0.849162	0.573725	0.450814
~Organizational resilience	0.460382	0.583131	0.808037	0.790206

For non-high innovative behavior, absence of organizational resilience and absence of thriving at work demonstrate strong necessary characteristics, revealing that lack of organizational support systems may be key factors inhibiting innovation. The relatively low consistency of trait mindfulness suggests that individual internal factors may need to interact with organizational environmental factors to effectively promote innovative behavior.

#### Analysis 3. Sufficient condition analysis

Using the established truth table to perform fsQCA analysis yields three types of solutions: simple, intermediate, and complex solutions. In standard logical analysis, comprehensive solutions would cover all potential condition combinations, but due to configuration diversity, such solutions can be quite complex and extensive. However, solving becomes difficult due to influencing factors, sometimes even resulting in unsolvable situations. Therefore, composite solutions are simplified into two categories: intermediate and simple solutions.

Simple solutions provide basic or minimal explanations, typically involving few key factors in fsQCA that directly and simply explain influences on phenomena or events. They help quickly understand relationships among main factors but may not cover all complexities. Intermediate solutions fall between simple and complex solutions, involving more factors and interactions but not covering all causal paths or interactions. They provide more comprehensive understanding than simple solutions while remaining relatively concise. Complex solutions offer the most thorough and comprehensive explanations, considering multiple factors and their complex interactions in fsQCA systems. They provide deep understanding, revealing multiple causal paths and complex interactive relationships, but may be more difficult to understand and interpret.

In fsQCA software, through Boolean minimization processing of truth table data, three different configurations affecting work performance were derived. These configurations are shown in Table 10, with consistency indices of 0.856465, 0.898496, and 0.930476 respectively, demonstrating high consistency levels. These three configurations provide a comprehensive perspective on how employee innovative behavior is influenced by key factors.

**Table 10:** Configuration pathways.

Core condition	configuration		
	P1	P2	P3
Trait mindfulness	●	○	●
Digital leadership	○	○	ⓧ
Thriving at work	○	●	●
Organizational resilience	●	●	○
Consistency	0.856465	0.898496	0.930476
Raw coverage	0.56074	0.74469	0.339379
Unique coverage	0.074105	0.258055	0.02142
Solution consistency	0.856725		
Solution coverage	0.840215		

● indicates condition present, ⓧ indicates condition absent, ○ indicates condition irrelevant (can be present or absent).

#### Overall analysis

The fsQCA intermediate solution reveals three significant causal pathways to innovative behavior, reflecting the configurational complexity and diversified characteristics of innovation formation. The overall solution coverage of 0.840215 indicates these pathways explain most conditions for innovative behavior variation; solution consistency of 0.856725 reflects reliable causal relationships between these pathways and innovative behavior. The three pathways maintain high consistency across complex, parsimonious, and intermediate solutions, indicating no logical redundancy.

The first pathway demonstrates high consistency with lower coverage, representing a relatively unique but reliable causal path. The second pathway shows higher consistency and coverage, indicating a more universally effective pathway. The third pathway, while having the highest consistency, has the lowest coverage, suggesting it may represent a highly efficient pathway under specific circumstances.

From a theoretical perspective, results support the “equifinality” principle in innovation formation, where different condition combinations can lead to identical outcomes, contrasting sharply with traditional linear models’ singular causal logic. The existence of three pathways also validates “configurational thinking” in complexity theory, focusing on variable combinations rather than independent effects of single variables.

#### Coverage analysis

Results show pathway two exhibits the highest raw coverage, indicating that synergistic effects of trait mindfulness and organizational resilience constitute the dominant configuration for promoting innovative behavior. This pathway explains most innovative behavior cases, reflecting universal applicability of this condition combination. Pathway one’s raw coverage of 0.56074, though lower, still demonstrates considerable explanatory power, suggesting that trait mindfulness and thriving at work can serve as effective innovation promoters even when organizational resilience is absent.

From unique coverage perspective, pathway two shows the highest unique contribution, with approximately 25.8% of innovative behavior cases explainable only through this pathway, highlighting the distinctive explanatory value of combining trait mindfulness with organizational resilience. Pathway one’s unique coverage of 0.074105 means approximately 7.4% of cases are explainable only by this configuration, representing an irreplaceable causal mechanism.

#### Individual variable analysis

The fsQCA analysis revealed distinct roles for each causal condition across the three identified pathways to high innovation. Trait mindfulness appeared in two pathways, demonstrating its foundational importance as an individual psychological resource. In the first pathway, it operates in conjunction with organizational resilience, revealing critical interactions between individual cognition and organizational support. Its absence from the second pathway supports the equifinality principle that multiple distinct mechanisms can produce equivalent innovative outcomes.

Digital leadership exhibited noteworthy configurational complexity by appearing in negated form (low digital leadership) in the third pathway (consistency = 0.339). This indicates that reduced digital leadership intervention does not necessarily inhibit innovation when employees possess sufficient individual psychological resources, specifically high trait mindfulness and thriving. This pattern aligns with self-determination theory’s emphasis on intrinsic motivation, challenging simplistic assumptions that digital leadership uniformly enhances innovation.

Thriving at work demonstrated configurational versatility by appearing in both the second and third pathways with different condition combinations. In the second pathway, thriving operates synergistically with organizational resilience, whereas in the third pathway, it combines with trait mindfulness under reduced digital leadership. According to broaden-and-build theory (Fredrickson, 2001), thriving states enhance positive emotions and broader cognitive repertoires that facilitate innovation exploration.

Organizational resilience appeared in both the first and second pathways through distinct mechanisms. In the first pathway, it synergizes with trait mindfulness to promote innovation (Lengnick-Hall et al., 2011), while in the second pathway, it combines with thriving at work. This dual presence underscores resilience as a foundational organizational condition that facilitates innovation both by amplifying individual psychological resources and by optimizing employees’ work states. These patterns reveal that innovation emergence follows complex, non-linear pathways where the same condition plays different functional roles depending on its configuration with other factors.

## Research conclusions and recommendations

### Main research conclusions

This chapter provides in-depth discussion of empirical analysis results, first explaining findings from SEM and fsQCA methods separately, then comparing their complementarity, and finally proposing managerial implications and future directions.

### Conclusions 1. SEM-based analysis conclusions

Structural equation modeling analysis shows trait mindfulness significantly promotes innovative behavior. High trait mindfulness employees, with stronger focus and openness, better perceive innovation opportunities and mobilize cognitive resources. The study validates two parallel mediation pathways: trait mindfulness affects innovative behavior through thriving at work (complete mediation), where high trait mindfulness employees experience stronger vitality and growth, motivating innovation investment; and trait mindfulness affects innovative behavior through organizational resilience (partial mediation), where trait mindfulness enhances organizational capacity to handle challenges. Digital leadership significantly negatively moderates the trait mindfulness-innovative behavior relationship, possibly due to conflicts between technology-driven management approaches and trait mindfulness’s present-moment focus.

### Conclusions 2. fsQCA-based analysis conclusions

The fsQCA analysis revealed three distinct configuration pathways leading to high innovative behavior. The first pathway combines trait mindfulness with organizational resilience, demonstrating that individual psychological resources complement environmental support in promoting innovation. The second pathway integrates thriving at work with organizational resilience, indicating that positive work experiences and organizational support jointly facilitate innovative behavior. The third pathway shows that under conditions of low digital leadership, the combination of trait mindfulness and thriving at work operates optimally to drive innovation. The overall solution achieved a coverage of 0.840 and consistency of 0.857, indicating that these three pathways collectively explain the substantial majority of innovative behavior generation in the sample. Organizational resilience appeared in both the first and second pathways through distinct mechanisms. In the first pathway, it synergizes with trait mindfulness to promote innovation (Lengnick-Hall et al., 2011), while in the second pathway, it combines with thriving at work. This dual presence underscores resilience as a foundational organizational condition that facilitates innovation both by amplifying individual psychological resources and by optimizing employees’ work states. These patterns reveal that innovation emergence follows complex, non-linear pathways where the same condition plays different functional roles depending on its configuration with other factors.

### Conclusions 3. Comparison and complementarity of methods

The structural equation modeling and fuzzy-set qualitative comparative analysis revealed different aspects of innovative behavior formation, demonstrating both consistency and clear differences that form effective complementarity. Regarding commonalities, both methods confirmed positive effects of trait mindfulness, thriving at work, and organizational resilience on innovative behavior, enhancing conclusion reliability. Both approaches also revealed complex effects of digital leadership on the trait mindfulness-innovative behavior relationship, with SEM showing negative moderation while fsQCA’s third pathway demonstrated that low digital leadership conditions favor innovation when combined with trait mindfulness and thriving.

The methods differed substantially in analytical focus and capabilities. SEM focused on linear relationships and causal paths, emphasizing direct and indirect effects of single factors using variable-oriented logic. In contrast, fsQCA adopted case-oriented configurational perspectives, emphasizing multi-factor combinations. While SEM cannot capture non-linear relationships and causal asymmetry, fsQCA explicitly considered these complexities, identifying multiple equifinal pathways and unique configurations such as the third pathway’s counterintuitive finding that low digital leadership can benefit innovation under specific conditions.

The complementarity between these methods provided richer insights than either approach alone. SEM established solid foundations for understanding direct relationships and mediation mechanisms, while fsQCA revealed how variables combine in different ways to influence innovation. Notably, SEM’s negative moderation finding received nuanced explanation through fsQCA’s third pathway configuration. This multi-method integration enabled focus on both general trends and case-specific patterns, grasping universal relationships while identifying unique pathways potentially masked by average effects. The combination enhanced conclusion robustness and provided more detailed, targeted guidance for organizational practice. Organizational resilience appeared in both the first and second pathways through distinct mechanisms. In the first pathway, it synergizes with trait mindfulness to promote innovation (Lengnick-Hall et al., 2011), while in the second pathway, it combines with thriving at work. This dual presence underscores resilience as a foundational organizational condition that facilitates innovation both by amplifying individual psychological resources and by optimizing employees’ work states. These patterns reveal that innovation emergence follows complex, non-linear pathways where the same condition plays different functional roles depending on its configuration with other factors.

### Managerial recommendations

Based on empirical findings, this study provides managerial implications and recommendations for both individual and organizational levels.

### Recommendations 1. Individual level

First, given trait mindfulness’s significant impact on innovative behavior, employees should consciously cultivate mindfulness capabilities. Specifically, employees can enhance present-moment awareness and acceptance through mindfulness meditation and focused breathing exercises. Research demonstrates positive effects even from brief mindfulness training (Ilies et al., 2024). Additionally, employees can participate in professional mindfulness workshops, systematically learn techniques, and integrate them into daily work through focused task completion and emotional awareness.

Second, since thriving at work mediates trait mindfulness’s effect on innovation, employees should enhance their thriving experiences. This includes setting appropriately challenging goals to increase vitality and actively seeking work meaning and value to cultivate passion and growth. Research shows employees viewing work as “calling” rather than “career” experience higher thriving levels (Schabram et al., 2023). Maintaining work-life balance is also crucial for sustaining thriving.

Third, given organizational resilience’s importance in innovation, employees should develop challenge-coping abilities. This includes cultivating positive attitudes toward uncertainty and change, learning effective stress management techniques, and maintaining openness to view setbacks as learning opportunities. Research indicates high-resilience employees not only better manage stress but also extract experience from difficulties, promoting long-term career development (Darvishmotevali, 2025).

### Recommendations 2. Organizational level

First, organizations should build mindfulness-oriented cultures through organizational training programs, inviting mindfulness experts or developing internal courses. Organizations can provide mindfulness resources and spaces (meditation rooms, app subscriptions) while integrating mindfulness into organizational values, encouraging focused, aware, and accepting work attitudes.

Second, organizations should create environments promoting thriving through: designing challenging and meaningful work content; establishing fair, transparent performance evaluation and promotion mechanisms; providing diverse career development and learning opportunities. Research shows perceived development opportunities significantly correlate with thriving levels (Sun et al., 2024).

Third, organizations should enhance resilience by establishing effective crisis management mechanisms, cultivating team collaboration, and creating open, inclusive atmospheres. Encouraging knowledge sharing and experience exchange are important pathways for strengthening organizational resilience. Research indicates high-resilience organizations not only better handle crises but also learn and grow from challenges, laying foundations for long-term innovation (Garrido-Moreno et al., 2024).

Finally, based on digital leadership’s negative moderation effect, organizations should carefully balance digital leadership application. While advancing digital transformation, they should emphasize human care and employee psychological needs, avoiding over-reliance on digital tools and data-driven decisions that neglect interpersonal interaction importance. For employees with different traits, organizations can create differentiated work environments—for instance, appropriately reducing digital intervention in certain innovation activities to create space for trait mindfulness. Organizations can also train leaders to effectively support employee innovation in digital environments, balancing technological application with interpersonal interaction.

### Recommendations 3. Research limitations and future directions

#### Research limitations

Despite valuable findings, this study has several limitations requiring consideration when interpreting results. Research design limitations include cross-sectional design restricting causal inference and inability to exclude reverse causality possibilities. Sample limitations involve data primarily from specific regions and industries, affecting generalizability, with insufficient consideration of potential moderating effects from different organizational cultures, job characteristics, and individual differences. Measurement limitations include potential failure to fully capture trait mindfulness’s multidimensional nature as a complex psychological construct, subjective bias in self-reported innovative behavior measurement, and possible neglect of confounding variables like personality traits and cognitive abilities. Methodological limitations, while combining SEM and fsQCA, include limited in-depth interpretation of fsQCA results, lack of qualitative evidence supporting deep mechanisms of pathways, and primary focus on individual level without sufficient consideration of team and organizational factors.

### Future research directions

Addressing these limitations, future research can expand in four areas:

Research design should adopt longitudinal designs collecting data at different time points to reveal causal relationships, combine mindfulness intervention experiments to verify influence mechanisms, and employ multi-source data collection to reduce common method bias; Variable exploration can subdivide different dimensions of trait mindfulness and differential effects of digital leadership, incorporate potential mediators like psychological safety to construct more complete theoretical models; Contextual expansion should validate models across diverse industries and organizational cultures, examine moderating effects of organizational size and team characteristics, particularly focusing on relationships between trait mindfulness and innovative behavior under high-stress contexts; Methodological innovation should integrate qualitative research to deeply understand fsQCA pathway mechanisms, apply social network analysis to explore team-level diffusion effects, and utilize multilevel linear modeling to construct cross-level theoretical models.

These expansions will help refine theoretical frameworks of trait mindfulness influencing innovative behavior and provide more precise guidance for organizational management.

## Data Availability

The original contributions presented in the study are included in the article/supplementary material, further inquiries can be directed to the corresponding author.
